# Microbial Populations of Stony Meteorites: Substrate Controls on First Colonizers

**DOI:** 10.3389/fmicb.2017.01227

**Published:** 2017-06-30

**Authors:** Alastair W. Tait, Emma J. Gagen, Sasha Wilson, Andrew G. Tomkins, Gordon Southam

**Affiliations:** ^1^School of Earth, Atmosphere and Environment, Monash University, Melbourne VIC, Australia; ^2^School of Earth and Environmental Sciences, The University of Queensland, St. Lucia QLD, Australia

**Keywords:** astrobiology, geomicrobiology, 16S rRNA gene, mars analog site, meteorites, Nullarbor Plain, arid soils

## Abstract

Finding fresh, sterilized rocks provides ecologists with a clean slate to test ideas about first colonization and the evolution of soils *de novo.* Lava has been used previously in first colonizer studies due to the sterilizing heat required for its formation. However, fresh lava typically falls upon older volcanic successions of similar chemistry and modal mineral abundance. Given enough time, this results in the development of similar microbial communities in the newly erupted lava due to a lack of contrast between the new and old substrates. Meteorites, which are sterile when they fall to Earth, provide such contrast because their reduced and mafic chemistry commonly differs to the surfaces on which they land; thus allowing investigation of how community membership and structure respond to this new substrate over time. We conducted 16S rRNA gene analysis on meteorites and soil from the Nullarbor Plain, Australia. We found that the meteorites have low species richness and evenness compared to soil sampled from directly beneath each meteorite. Despite the meteorites being found kilometers apart, the community structure of each meteorite bore more similarity to those of other meteorites (of similar composition) than to the community structure of the soil on which it resided. Meteorites were dominated by sequences that affiliated with the Actinobacteria with the major Operational Taxonomic Unit (OTU) classified as *Rubrobacter radiotolerans.* Proteobacteria and Bacteroidetes were the next most abundant phyla. The soils were also dominated by Actinobacteria but to a lesser extent than the meteorites. We also found OTUs affiliated with iron/sulfur cycling organisms *Geobacter* spp. and *Desulfovibrio* spp. This is an important finding as meteorites contain abundant metal and sulfur for use as energy sources. These ecological findings demonstrate that the structure of the microbial community in these meteorites is controlled by the substrate, and will not reach homeostasis with the Nullarbor community, even after ca. 35,000 years. Our findings show that meteorites provide a unique, sterile substrate with which to test ideas relating to first-colonizers. Although meteorites are colonized by microorganisms, the microbial population is unlikely to match the community of the surrounding soil on which they fall.

## Introduction

The Nullarbor Plain is a 20-million year old and ∼200,000 km^2^ area dominated by limestone karst that spans the southern regions of South Australia (SA) and Western Australia (WA) (Webb and James, 2006). It is a semi-arid environment characterized by an extreme average summer UV-index of 12.0 and a moderate average UV-index of 3.3 in the winter^1^. The Nullarbor Plain has high evaporation rates (2000–3000 mm/yr) with low rainfall (150–400 mm/yr)^2^ and occasional flooding on its flat topographic profile. This is a deflationary surface made up of aeolian sediments and a ∼1-m thick calcrete cap covers much of the region (Webb and James, 2006). The Nullarbor is named for its lack of trees; it is a sparse shrub-land dominated by the shrubs *Antiplex* and *Maireana*, which are colloquially known as ‘salt bush’ (Gillieson et al., 1994). The Nullarbor reached its present aridity ∼1 m.y.a (Webb and James, 2006) and the presence of evaporates in its cave systems indicates this aridity has been a stable climatic feature throughout the Pleistocene (Goede et al., 1992). Palynology and cave excavation also indicate that a period of prolonged aridity existed between 20 and 10 ka, at the end of the last ice age (Martin, 1973). Aridity has been a constant feature of this region, making the Nullarbor Plain one of the most homogenous terrains on the planet. Very few microbial studies have been done on the Nullarbor; although distally related research includes the ecology of cryptogrammic crusts from the region (Eldridge and Greene, 1994; Eldridge, 1998). Research more relevant to molecular studies includes analysis of novel chemolithoautotrophic microbial communities inside cave environments deep under the Nullarbor Plain (Holmes et al., 2001; Tetu et al., 2013). The microbial ecology of the Nullarbor topsoil remains unknown; however, microbial ecology studies of soils from other desert regions in Australia, such as the Sturt National Park, New South Wales, have been conducted using the 16S rRNA gene marker (Holmes et al., 2000). Holmes et al. (2000) found that a novel *Rubrobacter* species (a member of the Actinobacteria) dominated desert soil samples from that region at a relative abundance of 2.6–10.2%. Studies from the Atacama Desert have previously shown that *Acidobacteria* and *Proteobacteria* are less common in soils from hyperarid regions (Neilson et al., 2012). Although these two phyla are more abundant in forested and pastoral soils (Janssen, 2006), the Actinobacteria seem to dominate in arid environments.

One area of research tackling how communities develop over time is that surrounding “pioneer organisms” in fresh volcanic material (Englund, 1976; Kelly et al., 2014). The primary goals such studies are to identify the first organisms to colonize lava flows post eruption, and to follow changes in community structure with time (Kelly et al., 2010, 2011, 2014). Cooled lava flows represent sterile environments with which to test colonization hypotheses; however, new lavas commonly overprint past eruptive successions. Thus, given sufficient time, the pioneering communities of successive lavas [whilst initially different from those of past successions due to localized heterogeneities in the soil (Kelly et al., 2014)] will eventually increase their community diversity until the populations begin to look similar to the microbial populations of previous units. This process has also been observed in arctic soils (Schütte et al., 2010). Such studies raise the following questions about the role of a substrate in controlling the composition of its microbial community: (A) Is the endolithic microbial community controlled by the substrate [i.e., does the rock itself provide an environmental/nutritional advantage or does a level of ‘plasticity’ in microbial communities shape bulk rock environments into distinct microenvironments (Los Ríos et al., 2003)?]. (B) Is it inevitable that all rocks, independent of their elemental and mineralogical composition, converge on an ecological community ‘fingerprint’ characterized by an increase in species richness and structure over time within a given region? The latter case has been seen in Icelandic lava fields (Kelly et al., 2014). It is difficult to answer these questions in settings, such as lava flows, that produce sterile rocks of homogeneous composition. However, the introduction of sterile rocks into a non-sterile and petrologically different setting could be used to examine whether community structure is controlled by substrate composition or by stochastic processes. Ideally, such an experiment could be conducted over a long period of time (i.e., centuries to tens of millennia).

Here, we employ chondritic meteorites that have fallen to the limestone Nullarbor Plain over the past ∼35 thousand years (Jull et al., 2010) to test these ideas. Chondritic meteorites are sterile owing to their formation in the proto-planetary disk before the accretion of Earth (Minster and Allègre, 1979; Bennett and McSween, 2012). Meteoroids enter Earth’s atmosphere at speeds of 11.2–72.8 km/s (Ceplecha et al., 1998), compressing atmospheric gasses to produce a plasma that oblates the meteoroids to produce a ∼1-mm thick layer of molten silicate glass called a ‘fusion crust.’ This process is often preceded or followed by meteoroids experiencing one or more high-energy air blasts (Brown et al., 2013). Such conditions should destroy any microorganisms encountered in Earth’s upper atmosphere and render the meteorites sterile. During ‘dark flight’, in which bolide fragments fall at terminal velocity (>400 km/hr) through the troposphere, they may encounter atmospheric microorganisms. However, fallen meteorites continually interact with troposphere, which is the lowest layer of Earth’s atmosphere. Thus, atmospheric contamination of a meteorite during its fall to Earth is unlikely to have a significant effect on community development.

Chondrites are also mafic to ultramafic in composition, which provides a contrast in composition relative to the more common continental lithologies at Earth’s surface. Chondritic meteorites are similar in elemental and mineralogical composition to mafic rocks on Earth {e.g., they contain olivine [(Mg,Fe)_2_SiO_4_], plagioclase [(Na,Ca)(Si,Al)_4_O_8_], and enstatite [Mg_2_Si_2_O_6_] (Dunn et al., 2010)}; thus, results of first colonizer studies on chondrites can be directly compared to previous results from volcanic settings.

Lastly, meteorites contain troilite [FeS] and FeNi alloys [Fe_1-x_Ni_x_] that can be used as electron donors by iron and sulfur oxidizing organisms (e.g., *Acidithiobacillus ferrooxidans*). This provides a suitable contrast to the fossiliferous limestone of the Nullarbor Plain, which predominantly contains calcite [CaCO_3_] and quartz [SiO_2_] (Webb and James, 2006). In this study, 16S rRNA gene analysis was used to assess which of the microorganisms that have adapted to soils over the Nullarbor limestone can colonize chondrites. By examining the bacterial and archaeal populations within Nullarbor Plain soil and meteorites overlaying this soil, we shed new light on whether the structure of microbial communities in meteorites is determined by geochemical and niche factors (i.e., the composition and properties of the meteorites themselves) or by broader environmental factors operating in the Nullarbor Plain.

## Materials and Methods

### Field Sampling

Samples of meteorites and soil were collected on two consecutive days in 2015 during Monash University’s annual expedition to the Nullarbor Plain. All meteorites, soil samples and thin sections are curated in the collection of the School of Earth, Atmosphere and Environment at Monash University.

**FIGURE 1:**
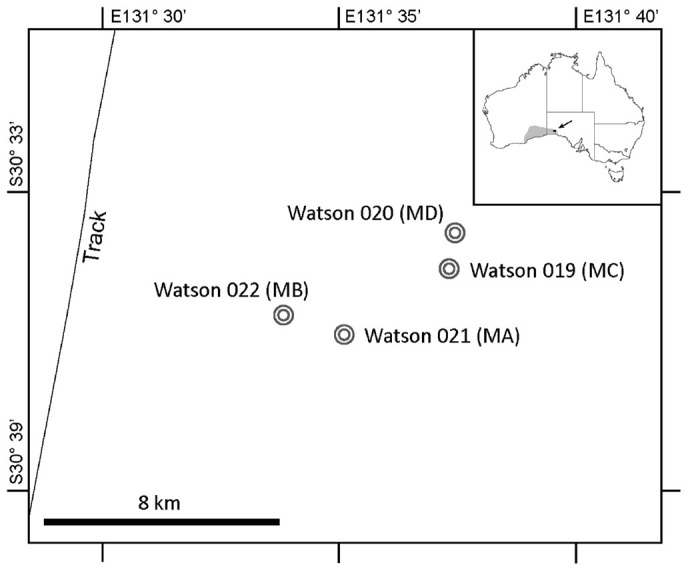
Nullarbor Plain Map. This figure shows the Watson location in the Nullarbor Plain, Australia. Circles indicate the locations from which the four meteorites and associated soils were collected.

A total of four meteorites and associated soils were collected from two search locations that are ∼8 km apart within the Nullarbor Plain, Australia (**Figure 1**). The two meteorites found at each search location (roughly ∼1.4 km apart in both cases) were collected aseptically for microbial community/diversity analysis. The soil from directly beneath each meteorite was also collected in this manner. The properties of topsoil varied significantly between sample sites. Soils adjacent to meteorites were characterized by either (1) cryptogrammic surfaces (Eldridge and Greene, 1994) or (2) deflationary gibber surfaces, which are soils covered in a pavement of limestone pebbles and the occasional meteorite. Sampling cryptogrammic surfaces would have artificially inflated the representation of prokaryotes associated with lichens in soil samples, where as sampling gibber surfaces would have artificially underrepresented the number of phototrophs and xerophiles. Ultimately, soil samples were collected from beneath the meteorites to create a uniform sampling method, although this may have resulted in underrepresentation of xerophiles and phototrophs compared to other soils in the region.

**FIGURE 2:**
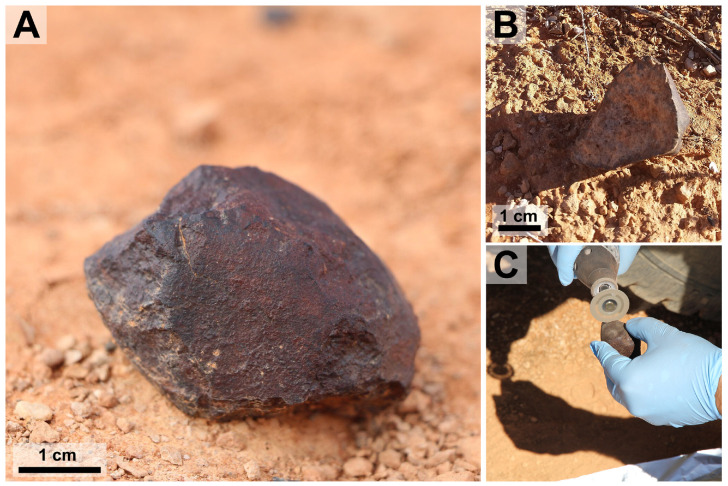
Meteorite Samples. This figure shows two meteorites used in the experiment and depicts field-based sub-sectioning methods. **(A)** Sample *Watson 021 in situ*. **(B)** Sample *Watson 019 in situ* after being flipped over during collection. **(C)** Sample *Watson 019* being sub-sectioned over autoclaved aluminum foil.

A sterile 10 mL centrifuge tube was used to collect a short push-core from the upper ∼2 cm of the soil directly underneath each meteorite. We anticipated that soils collected from beneath meteorites would provide an analog to the environmental conditions inside meteorites (i.e., low light flux and low evaporation). The meteorites were sub-sectioned in the field using a diamond-embedded dermal saw that was washed in 70% ethanol and an effort was made to avoid sectioning meteorite surfaces covered in soil (**Figure 2C**). Sub-sectioning was done to expose cryptoendolithic and chasmoendolithic microbial communities while minimizing post-collection contamination. Any contaminant minerals or microorganisms introduced during processing in the field would most likely be indigenous to the Nullarbor Plain. Meteorites were handled as little as possible using nitrile gloves washed in 70% ethanol and the sub-sectioned meteorites were cut over autoclaved aluminum foil and deposited in sterile 50 mL centrifuge tubes that were sealed with paraffin film. Centrifuge tubes containing soil samples were also sealed with paraffin film. Both the meteorite and the soil samples were snap frozen in the field using a liquid-nitrogen dry shipper and transported frozen to the laboratory where they were stored at -20°C before DNA extraction.

### Meteorite Sample Description

Meteorites were classified according to the rubric of Van Schmus and Wood (1967). Shock classifications were determined using Stoffler et al. (1991) and weathering classifications were obtained using the rubric outlined in Wlotzka (1993).

*Watson 019* is a fragment of a L6 ordinary chondrite weighing 83 g. This single stone was found ‘face down’ with a fully intact fusion crust only on the side that did not face the ground (**Figures 2B,C**). The sample is relatively unweathered, exhibiting a (W1) weathering profile, and shows signs of moderate shock including cross cutting melt veins (S3). *Watson 020* is a L5 ordinary chondrite that was found in three fragments, with a total mass of 134 g, over an area of ∼20 m^2^. The fragments were relatively fresh, showing a W1 weathering profile but minimal remaining fusion crust. *Watson 020* fragments have light shock textures (S2). The fragment chosen from *Watson 020* for 16S rRNA gene analysis contains a large vein of alteration minerals that cross cuts the sample. *Watson 021* is a single H7 ordinary chondrite, weighing 135 g (**Figure 2A**). The sample has a large crack, lined with alteration minerals, that runs down its middle and only one third of its fusion crust remains intact. This meteorite shows near complete oxidation of its reduced metal and sulfide phases making this a W4 chondrite. *Watson 021* is extensively shocked (S4), exhibiting globular silicate metal emulsions and shock veins that crosscut the sample. *Watson 022* is a single, 11.1-g oriented L6 ordinary chondrite. This sample shows extensive silicate/metal emulsions and crosscutting silicate veins indicating extensive shock (S4). The metal and troilite have experienced light weathering (W2).

The meteorite names used above are provisional, and subject to change. Meteorite classifications have been sent to the Meteoritic Bulletin and they await official naming and cataloging. All samples of meteorites and soils described in this study have been given a two-character sample ID for ease of reference throughout this manuscript. Meteorites are given the prefix ‘M,’ whereas soils are given the prefix ‘S’ (see **Table 1** for naming details).

**Table 1:** Sample list and alpha diversity.

Sample Name^ℵ^	Group ID	Type	Shock	Weathering	Nseqs	Sobs^∗^	Coverage^∗^	Chao^∗♢^	Inv. Simpson^∗♢^	Shannon^∗^
Watson 021	MA	L7	S4	W4	34871	2051 (N/A)	0.9704 (N/A)	3961 (N/A)	22.77 (N/A)	4.734 (N/A)
Watson 022	MB	L6	S4	W2	49758	2181 (19)	0.9626 (0.0006)	4935 (177)	23.7 (0.19)	4.592 (0.007)
Watson 019	MC	L6	S3	W1	62811	2702 (23)	0.9631 (0.0007)	5233 (177)	29.36 (0.34)	5.390 (0.009)
Watson 020	MD	L5	S2	W1	55066	8955 (46)	0.8044 (0.0015)	33057 (837)	57.28 (0.77)	6.747 (0.010)
Soil (Watson 021)	SA	Soil	–	–	–	–	–	–	–	–
Soil (Watson 022)	SB	Soil	–	–	38159	3144 (12)	0.9441 (0.0004)	7699 (156)	24.75 (0.10)	4.915 (0.004)
Soil (Watson 019)	SC	Soil	–	–	57753	12845 (54)	0.6973 (0.0017)	68819 (1679)	101.91 (1.66)	7.616 (0.010)
Soil (Watson 020)	SD	Soil	–	–	60281	15272 (57)	0.6316 (0.0018)	88505 (2075)	342.71 (5.45)	8.190 (0.009)

^♢^ *Student’s *t*-test was conducted between the meteorite and the soil substrates on Inv. Simpson with a *p* > 0.05 for both. Standard deviations are given in brackets.*

*^∗^Samples were subsampled to the library size of MA, which had the lowest number of sequences: 34871. ^ℵ^Names of all meteorites are still waiting official classification by the Meteoritical Bulletin*.

### DNA Extraction and Sequencing

DNA was extracted from the meteorite sub-sections and soil samples using a bead-beating cetyltrimethylammonium bromide (CTAB) based method coupled with column-based purification of nucleic acids using a PowerSoil^®^ DNA isolation kit (MO BIO Laboratories Inc., Carlsbad, CA, United States) as per the manufacturer’s protocols (Gagen et al., 2010, 2013). Less than 20 ng of DNA extracted from each sample was used as a template in a 50 μL PCR reaction to amplify the V6–V8 region of the 16S rRNA gene using primers 926f and 1392r (Engelbrektson et al., 2010). These primers target the domains Bacteria and Archaea and contain the Illumina specific adapter sequences (adapter sequences in capitals): 926F: 5′-TCGTCGGCAGCGTCAGATGTGTATAAGAGACAGaaactyaaakgaattgacgg-3′ and 1392wR: 5′-GTCTCGTGGGCTCGGGTCTCGTGGGCTCGGAGATGTGTATAAGAGACAGacgggcggtgtgtrc-3′. Libraries were prepared as outlined by Illumina (#15044223 Rev B) except that Q5 Hot Start High-Fidelity polymerase and PCR mastermix were used (New England Biolabs, Ipswich, MA, United States). PCR amplicons were purified using Agencourt AMPure XP beads (Beckman Coulter, Brea, CA, United States). Purified DNA was indexed with unique 8 bp barcodes using the Illumina Nextera XT v2 Index Kit sets A-D (Illumina, San Diego, CA, United States) and the same PCR mastermix as previously. Indexed amplicons were pooled together in equimolar concentrations and sequenced on a MiSeq Sequencing System (Illumina) using paired-end sequencing with MiSeq Reagent Kit v3 (600 cycle) (MS-102-3003, Illumina) in accordance with the manufacturer’s protocol at the Australian Centre for Ecogenomics, The University of Queensland. Sequences have been submitted to the National Centre for Biotechnology Information Sequence Read Archive and can be accessed using the accession number SRP100888, or the BioProject number PRJNA377370.

### Sequence Processing

Processing of DNA sequence data was done using MOTHUR v1.38.1 (Schloss et al., 2009) and only forward reads were used for analysis. Sequences were trimmed based on the quality score using a ‘qwindowaverage’ of 35, across a sliding window of 50, after which the PCR primer was removed. Sequences were trimmed to 250 nt and any sequences shorter than 250 nt, or containing ambiguous bases and/or homopolymers in excess of 8 nt were also cut. Further sequence analysis was done as per Kozich et al. (2013), accessed online October 2016. The Silva reference database v123 (Quast et al., 2013) was used for taxonomic classification and alignment of sequences. Putative chimera were determined using UCHIME (Edgar et al., 2011) in MOTHUR (Schloss et al., 2009) and the Silva Gold reference database v123 (Quast et al., 2013) and were removed from further analysis. Anomalous taxa including Eukaryota, unknown classification, mitochondria, and chloroplasts, were also removed from the dataset. Sequences were clustered into OTUs (Operational Taxonomic Units) at a distance of ≤0.03.

### Sequence Analysis

**FIGURE 3:**
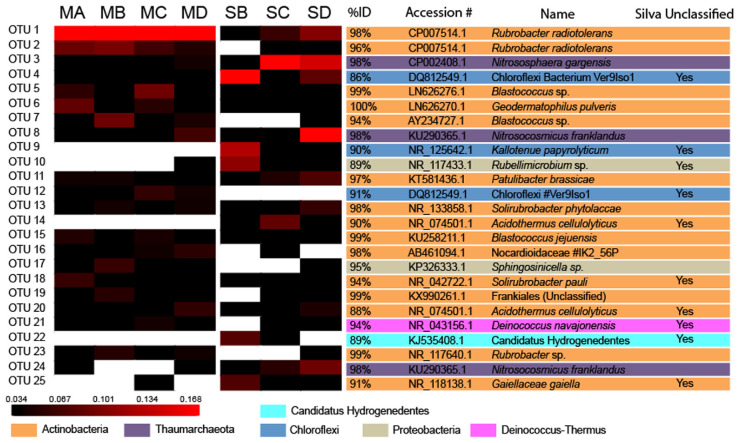
Heatmap of Operational Taxonomic Unit Abundance. Heatmap analysis of OTU abundance in meteorites and soil samples. Analysis performed for OTUs at a distance of ≤0.03. The scale bar represents the fractional abundance of each OTU within each sample. The identity score, accession number, and the name of the nearest named isolate according to NCBI BLAST are indicated beside the heatmap. OTUs that were unable to be classified beyond the level of the domain Bacteria in the *Silva* database are also noted.

Representative sequences from the most abundant 25 OTUs (**Figure 3**) were compared to publicly available sequences using Basic Logical Alignment Search Tool (BLAST) at the National Centre for Biotechnology Information (NCBI) excluding uncultured and environmental organisms. After 25 OTUs there were no abundant OTUs of interest, thus for the sake of brevity only the most abundant OTUs are discussed in detail. The dataset was subsampled 1000 times to the size of the smallest library to normalize the data before analysis^3^ (Van Horn et al., 2016; Jiang and Takacs-Vesbach, 2017). Further alpha and beta diversity analysis was conducted using MOTHUR (Schloss et al., 2009) as per the method in Kozich et al. (2013). Additional multivariate data analysis was conducted between the meteorite and soil substrates using Analysis of Molecular Variance (AMOVA) and Non-metric Multidimensional Scaling (NMDS) (Excoffier et al., 1992) in MOTHUR. In order to explore which species could be used as a biomarker for each substrate type (e.g., meteorite vs. soil), we used Linear Discriminant Analysis Effect Size (LEfSe) (Segata et al., 2011). Student’s *t*-test was used to assess the difference in relative abundance, at phylum and class level, between meteorites and soils. Lastly, Silva classifications were searched for metal/sulfur cycling affiliated organisms.

## Results

### Major OTU Classification

**FIGURE 4:**
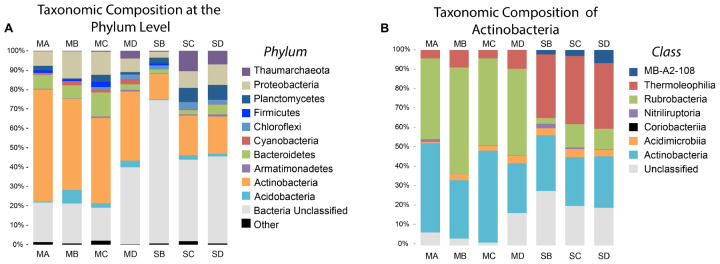
Phyla and Actinobacteria Abundance of Meteorites and Soil. This figure shows the relative abundance of different phyla and classes. **(A)** The major phyla classified according to the Silva taxonomy identification. ‘Other’ phyla include all phyla present at abundances less than 3%. ‘Bacteria Unclassified’ were OTUs that could not be classified below the domain Bacteria. **(B)** Taxonomic composition of the important soil phylum, Actinobacteria.

After data processing a total of 358,699 sequences across all samples were grouped at a distances of <0.3, which resulted in 57,431 unique OTUs of which 44,254 were singleton OTUs. DNA was recovered from all samples except for one of the soils, SA. Of the top 25 OTUs in all samples (**Figure 3**) only three were identified as Archaea. These were classified as *Thaumarchaeota*, a phylum that contains all the known Ammonia Oxidizing Archaea (AOA) (Pester et al., 2011). These were major OTUs in the soil samples but present only at low abundance in the meteorite samples (**Figure 4**). They demonstrated 98% 16S rRNA gene identity to the known ammonia oxidizing Archaea, *Nitrososphaera gargensis* (OTU3) and *Nitrosocosmicus franklandus* (OTU8 and OTU24) (Hatzenpichler et al., 2008; Lehtovirta-Morley et al., 2016).

The dominant OTU in the meteorite samples (OTU1) demonstrated 98% 16S rRNA gene identity to *Rubrobacter radiotolerans – #*CP007514.1. This organism is an aerobic, heterotrophic thermophile (30–55°C) (Egas et al., 2014). We found this OTU to represent 27.3% ± 8.7% of total sequences in the meteorites, whereas it comprised only 1.6% ± 1.1% of the total abundance of sequences in soils. Another dominant OTU, OTU2, was present in all meteorites but was only found at low abundance in two of the soil samples (SC and SD). OTU2 shared 96% 16S rRNA gene identity with *R. radiotolerans*.

The availability of FeNi-alloys and troilite in the meteorites presents an opportunity for biogenic metal/sulfur cycling. As such, we used the Silva classification to search for common genus members that are known to contain species capable of iron or sulfur cycling metabolisms. We searched for: *Acidithiobacillus, Anaeromyxobacter, Caldivirga, Desulfovibrio, Gallionella, Geobacter, Leptospirillum, Shewanella, Sideroxydans, Sphaerotilus*, and *Thiobacillus*. Our search returned members of *Geobacter* (8 non-singleton unique OTUs) and *Desulfovibrio* (13 non-singleton unique OTUs). Out of these OTUs, the most abundant OTU was OTU71. The nearest named isolate to OTU71 was *Geobacter anodireducens -* #CP014963.1 (100% 16S rRNA gene identity across the region sequenced). *G. anodireducens* is able to reduce Fe(III) and sulfur with acetate as the electron donor (Sun et al., 2014, 2016). The most common *Desulfovibrio* was OTU501. The nearest named isolate to OTU501 shared 100% 16 rRNA gene identity across the region sequenced with *Desulfovibrio desulfuricans –* #KU921226.1, strains of which are known to reduce sulfate (Mangalo et al., 2007). Refer to Supplementary Table 1 for a full list of possible iron/sulfur cycling organisms found in this study. This list is not exhaustive and the 16S rRNA gene analysis is not a functional analysis of possible metabolisms. It is possible that some of the other species may cycle metal or sulfur, but it is outside the ability of this technique to discern.

### Alpha Diversity Indices

**FIGURE 5:**
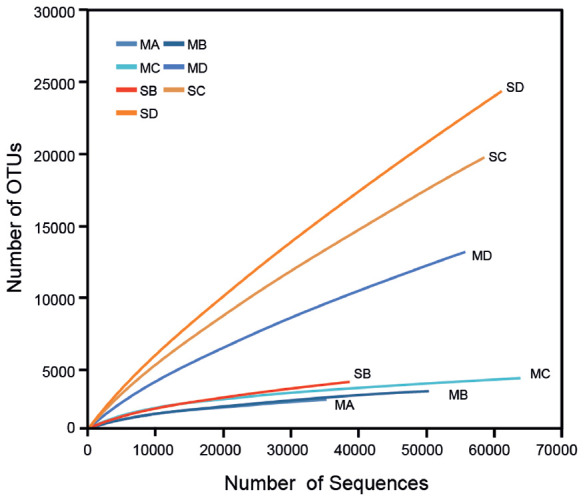
Rarefaction Curve. Rarefaction analysis of all samples at a clustering distance of ≤0.03. Warm colors are soil samples, cool colors are meteorites.

Rarefaction analysis of 16S rRNA gene libraries clustered at <0.03 indicated that meteorite samples were sequenced with sufficient coverage (i.e., the rarefaction curves reach plateaus), with the exception of meteorite MD that did not plateau, indicating partial coverage for that sample. Meteorite sample MD was similar to the soils whose rarefaction curves also did not plateau, indicating further sequencing for the soil samples would be needed, with the exception of SB (see **Figure 5**). Good’s coverage estimate for the percentage of species represented in a sample was generally higher for the meteorites (80–97%) than for the soils (63–94%) with the exception of SB and MD (see **Table 1**). The Chao species richness estimator predicted a much higher uncovered richness in the soils than the meteorite samples, with the exception of SB (see **Table 1**).

As indicated by the inverse Simpson index and Shannon index, there was generally greater species evenness in the soils than in the meteorites (see **Table 1**). Although species evenness in sample SB was considerably lower than that in the other two soils and sample MD, it showed markedly higher species evenness than the other three meteorite samples.

### Beta Diversity

The use of Non-parametric Analysis of Molecular Variance (AMOVA) (Excoffier et al., 1992), using the Yue and Clayton (2005) index for community structure between the soils and the meteorite, confirmed that microbial community structure was different between the meteorites and the soils (*p* < 0.05).

**FIGURE 6:**
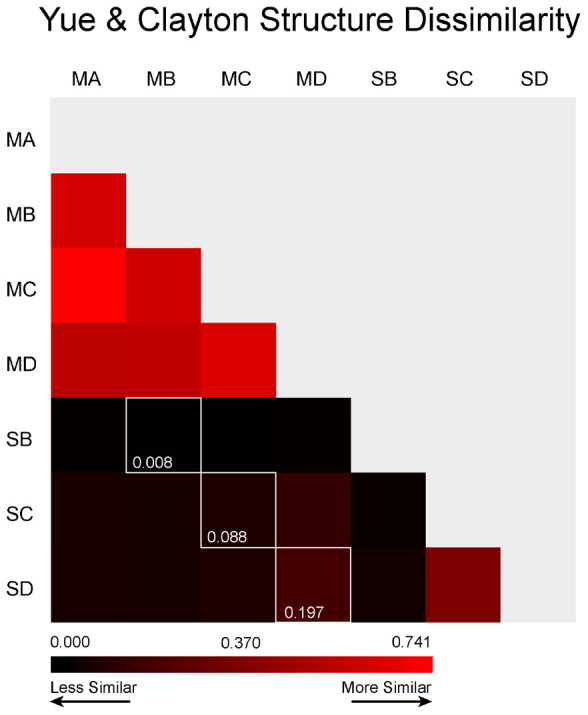
Community Structure Dissimilarity. This calculation was made using Yue and Clayton (2005) indices at a clustering distance of ≤0.03. The colors are scaled to the highest level of similarity between any two samples (red) and the lowest level of similarity (black). The white outline represents the direct comparison between the meteorite and its underlying soil.

**FIGURE 7:**
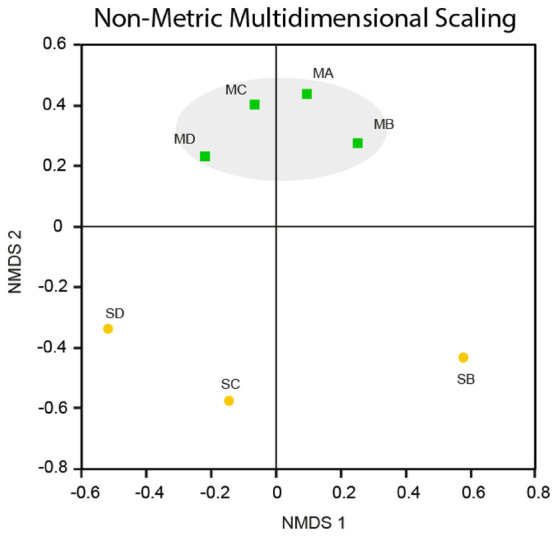
Non-metric Multidimensional Scaling. NMDS plot for Nullarbor meteorite (MA, MB, MC, MD) and soil (SB, SC SD) samples based on the Yue and Clayton (2005) community structure. The stress value is 0.146 [Stress values <0.2 indicate that an NMDS ordination plot has good spatial representation of differences between communities (Levshina, 2015)].

The meteorites had community structures that were much more similar to each other, whereas those in the soils displayed more variation amongst themselves (see **Figure 6**). This was confirmed by NMDS analysis (Borg and Groenen, 2005), which revealed that the meteorite samples clustered together, away from each of the soil samples, which did not cluster closely to each other (**Figure 7**). We ran a Spearman’s rank correlation coefficient analysis to establish which OTUs defined the two NMDS axes. The five major OTUs contributing to separation for axis NMDS 1 were: OTU8 (98% identity to *Nitrosocosmicus franklandus*) *p* = 0.018 for NMDS 1, OTU22 (89% identity to an uncultured *Candidatus Hydrogenedentes*, #KJ535408) *p* = 0.021 for NMDS 1, OTU25 (91% identity to *Gaiellaceae gaiella*) *p* = 0.021 for NMDS 1, OTU10 (89% identity to *Rubellimicrobium* sp. *p* = 0.021 for NMDS 1, and OTU9 (90% identity to *Kallotenue papyrolyticum*) *p* = 0.023 for NMDS 1. For axis NMDS 2, the top five OTU contributions were: OTU1 (98% identity to *Rubrobacter radiotolerans*) *p* =< 0.001 for NMDS 2, OTU21 (94% identity to *Deinococcus navajonensis*) *p* = 0.002 for NMDS 2, OTU2 (96% identity to *Rubrobacter radiotolerans*) *p* = 0.005 for NMDS 2, OTU24 (98% identity to *Candidatus Nitrosocosmicus franklandus*) *p* = 0.018 for NMDS 2, and OTU18 (94% identity to *Patulibacter* sp.) *p* = 0.031 for NMDS 2. None of the OTUs associated with metal/sulfur cycling genera appeared to have a strong influence on the NMDS separation.

### OTU Abundance

Actinobacteria accounted for 44.8% ± 9.4% of the total number of identified sequences in the meteorite samples. This was by far the most abundant phylum identified in the meteorites. The next most abundant phyla in the meteorite samples were Proteobacteria (10.0% ± 3.5%), Bacteroidetes (7.6% ± 3.9%), and Acidobacteria (3.4% ± 2.7%), with other phyla representing <2.0% each. Actinobacteria were dominant in the soils from our study (17.7% ± 3.9%), albeit to a lesser extent than in the meteorites, followed by Proteobacteria (7.4% ± 3.9%), Planctomycetes (6.0% ± 2.5%), and Thaumarchaeota (5.9% ± 5.2%), with other phyla present at <3.0% each.

The soils had a greater average abundance of unclassified bacteria (53.7% ± 17.6%) compared to the meteorites (23.6% ± 10.3%). BLAST analysis revealed that these “unclassified bacteria” were members of various phyla. Nine of these unclassified bacteria were represented in the most abundant 25 OTUs and overall they had poor identity scores compared to those OTUs that were identified from phylum or better. Two OTUs (OTU25 and OTU18) recorded poor identity scores with both Silva and BLAST. OTU25 was only distantly related (91% 16S rRNA gene identity) to the nearest named isolate, *Gaiella occulta* (Albuquerque et al., 2011), and demonstrated 96% identity to an uncharacterised organism that has been isolated from soil previously (Davis et al., 2011) and that is referred to as #Ellin7545. OTU18 was also only distantly related (94% 16S rRNA gene identity) to the nearest named isolate, *Solirubrobacter pauli* (Furlong et al., 2002) (**Figure 3**). Different classes within the Actinobacteria are found at greater abundance in the meteorites and soils. Rubrobacteria dominated in the meteorites (46.2% ± 5.8% of the Actinobacteria in meteorites) whereas the class Thermoleophilia were dominant amongst the Actinobacteria in the soils (33.7% ± 1.2%) (**Figure 4B**).

### OTU Substrate Associations

We used the student *t*-test to explore the relative abundance differences between phylum and classes of the particular substrates. We found that the presence of Actinobacteria associated strongly with the meteorites (*p* = 0.005) while Cyanobacteria were poorly associated with meteorites (*p* = 0.068). The strongly associated phyla differed in the soils: Planctomycetes (*p* = 0.032) and a poor association from Chloroflexi (*p* = 0.066). The phyla that did not associate well with either meteorites or soil were Armatimonadetes (*p* = 0.488), Firmicutes (*p* = 0.245), Acidobacteria (*p* = 0.285), and Proteobacteria (*p* = 0.379). Rubrobacteria, Thermoleophilia and the candidate class, MB-A2-108, were significantly different between the meteorites and the soils (*p*-values < 0.001, <0.001, and 0.021, respectively). Rubrobacteria was the dominant class of Actinobacteria in the meteorites, whereas Thermoleophilia and MB-A2-108 were more strongly associated with the soils.

We used Linear Discriminant Analysis Effect Size (LEfSe) analysis to investigate which indicator species were associated with the meteorites or soils (Segata et al., 2011). We found that most species did not associate well (i.e., *p* > 0.05) with either the soil or the meteorites. This also included all the OTUs affiliated with metal/sulfur cycling isolates (Supplementary Table 1). However, there were some notable exceptions in the top 25 OTUs used in previous beta diversity analysis. OTU1 and OTU2 (*R. radiotolerans*) associated to the meteorites (*p* = 0.034), as did OTU5 (*Blastococcus* sp.) *p* = 0.034, OTU6 (*G. pulveris*) *p* = 0.034, OTU12 (Chloroflexi) *p* = 0.028, OTU15 (*B. jejuensis*) *p* = 0.034, OTU19 (Frankiales) *p* = 0.034, OTU21 (*D. navajonensis*) *p* = 0.034, and OTU23 (*Rubrobacter* sp.) *p* = 0.032. The OTUs associated with the soils were OTU4 (Chloroflexi) *p* = 0.034, OTU9 (*K. papyrolyticum*) *p* = 0.019, OTU14 (*A. cellulolyticus*) *p* = 0.019, and OTU25 (*G. gaiella*) *p* = 0.028 (Supplementary Table 1).

## Discussion

### Meteorite Colonization

Alpha diversity analyses (**Table 1**) indicate that the meteorites have poor species richness and evenness, suggesting that they have been colonized by a few successful niche organisms. This has also been observed in microbial communities of ignimbrites (a volcanic deposit) in the hyper-arid Atacama desert (Wierzchos et al., 2013a). It would appear that species richness and structure is small in environments that select for multi-extremophiles. This is important for meteorites as they share many overlapping physical and chemical characteristics with ignimbrites, which likely lend themselves to the same style of initial colonization by microorganisms.

When a meteorite becomes a resident of the Nullarbor Plain it is colonized by environmental organisms derived (presumably) from soil as indicated by crossover species in the heat map (**Figure 3**). However, the soils have much greater species richness than the meteorites, indicating the microenvironment of the meteorites is unsuitable for some indigenous microbes. Indeed, the rarefaction curves (**Figure 5**) indicate that there considerable diversity within the soils that has not been accounted for whereas rarefaction curves for the meteorites generally plateaued.

Our AMOVA results show that, in spite of the presence of crossover species, the microbial community structures in meteorites and soils were significantly different, whereas all of the meteorites shared similar community structures (**Figure 6**). We attribute this difference in the soil to the establishment of distinctly different microenvironments within these samples. One caveat is that the soil samples were obtained from directly underneath the meteorites; such samples may have retained more moisture and experienced less environmental stress than soils that were more exposed to the atmosphere, allowing for a greater diversity of epilithic microorganisms. Despite the bias our sampling strategy may have introduced, there was still a large structural variation not only between the meteorites and the soils, but also between each of the soils. Thus, the lower structural variation reflected between the meteorite samples is probably due to chemical and/or physical homogeneity of the meteorites. The communities within soil and meteorite samples may have been similar initially, but are now structural different due to selective pressures (both geochemical and environmental). The meteorites in this study are chemically quite similar (all of our samples are L type ordinary chondrites), but they have quite varied physical characteristics. This would indicate that weathering, shock and even the degree of thermal-metamorphism that chondritic meteorites have undergone have little control on the composition of the microbial communities that inhabit them. Nonetheless, as previously discussed, ordinary chondritic meteorites are elementally and mineralogically quite homogeneous (Dunn et al., 2010), which probably explains the low variability in microbial communities in the meteorites compared to Nullarbor soils and previous studies of volcanic rocks (Kelly et al., 2010, 2011).

Weathering grade has been calibrated to the residency age of meteorites on the surface of Earth using radiometric dating methods (Al-Kathiri et al., 2005). Importantly, weathering grade and radiometric dates do not always correlate well for Nullarbor meteorites (Jull et al., 2010). Thus, the duration of residency of our four meteorite samples on the Nullarbor Plain, and the length of time available for microbial community structure to develop, cannot be determined from weathering grade alone (although, it would be possible to make reliable estimates in other deserts where weathering grade correlates strongly with residency age). Weathering grade does provide a relative estimate of the amount of oxidative weathering that has occurred in Nullarbor meteorites. However, our findings indicate that the current redox environment of the different meteorites does not result in a significantly different community structure (**Figure 6**). Tied intrinsically to the weathering grade of meteorites is their porosity. This porosity provides a range of microhabitats that could cater to microorganisms adapted to a range of different pH conditions (Tait et al., 2017), which could make them good substrates for a variety of microorganisms. However, we did not see any correlation with weathering or shock (two physical traits known to affect porosity). Meteorites are, however, dark in color, resulting in a contrast in albedo compared to the white limestone and lightly colored soil of the Nullarbor Plain, which could change the ambient temperatures of the rock (Wierzchos et al., 2013b). Such dark meteorites may support the survival of microorganisms in cold environments such as Antarctica, and could play a similar role for putative life in other hostile environments within our solar system, such as at the surface of Mars. However, in the desert on Earth, a dark rock could reach thermally restrictive temperatures for mesophiles. It may be that thermophilic microorganisms, may be exploiting this thermal niche in the meteorite to obtain almost exclusive access to the films of water that form on hygroscopic alteration minerals (Tait et al., 2017). These minerals are the direct product of weathering in meteorites and are not found in abundance within the Nullarbor soil. Given the different weathering and shock grades of the meteorites we expected to see more scatter in the community structure of the meteorites, however, this was not observed (**Figure 6**). In the absence of clear porosity driven separation in the meteorite community structure, other physical/chemical controls must be taking effect (i.e., hygroscopic mineral production, availability of native copper, fragmentation rate, competition with lichens). We interpret that it is the chemical homogeneity of the meteorites (i.e., they are all L type chondrites) and not their physical characteristics that drives the development of similar community structures for these meteorites.

It is possible that there is a nutritional advantage that causes the development of similar community structures in meteorites. Given the abundance of FeNi-alloys and FeS in these samples, the possibility of metal-cycling organisms was one that we explored. We found OTUs associated with known genera that cycle iron and sulfur in both the soil and the meteorites. However, none of these OTUs was significantly associated with the meteorites (Supplementary Table 1). The most abundant OTUs with similarity to known iron and sulfur cycling species were OTU71, and OTU501, which were most similar to known iron/sulfur and sulfur reducers, respectively. If the organisms forming these OTUs are indeed capable of iron and/or sulfur reduction, some abiotic oxidation and weathering of the meteorite would be required before these organisms could reduce the oxidized metals and sulfate, as most of the sulfur and iron in the meteorites is initially present in reduced phases (i.e., as Fe^0^, Fe^2+^, and S^2-^). These OTUs were not abundant in the libraries, and did not contribute significantly toward separation in the NMDS analysis, suggesting they are not key drivers shaping the community structure.

The iron and sulfur reducing organisms that are most similar to OTU71, and OTU501 are chemoorganotrophs. As such, some consideration should be given to discussing sources of organic carbon that are available to these organisms in the meteorites. The first possibility is that organic carbon is coming from other organisms. Many of the meteorites were found sitting on cryptogrammic crusts, making the decay of lichens and associated algae the largest and most likely source of organic carbon accessible to these microorganisms. Indeed, we have found meteorites that are covered lichen in the Nullarbor. The other possibility is that these organisms obtain organic carbon from an exogenic source, such as the meteorite itself. Ordinary chondrites, such as those in this study, contain little organic matter. Given their history of thermal metamorphism, most carbon will have been devolatilised on the parent body or turned to kerogen or graphite. Nonetheless, Polycyclic Aromatic Hydrocarbons (PAH) and amino acids can still be found in small concentrations in ordinary chondrites (Zenobi et al., 1992).

Contrastingly, carbonaceous chondrites, which were not examined as part of this study, typically have not undergone high degrees of thermal metamorphism. Carbonaceous chondrites contain more organic material than ordinary chondrites. The Murchison CM2 meteorites organic inventory includes 1.45 wt% of macromolecular organic compounds and acetate in concentrations >300 ppm, as well as other organic compounds that could support heterotrophic metabolisms (Sephton, 2002). The carbon content of carbonaceous meteorites has been shown to support microorganisms under laboratory conditions (Mautner, 1997). Carbonaceous meteorites also contain native Fe(III) phases (Rubin, 1997), meaning that some of the metal cycling organisms affiliated with the OTUs found in this study could potentially use the native acetate and Fe(III) phases found within carbonaceous meteorites. Moreover, they would have no need for input of terrestrial organic matter nor would they have to wait for the meteorite to start weathering for Fe(III) to become available.

### Comparison to Other Soils

The microbial communities in Nullarbor soil samples differ from those commonly found in pastoral and forested soils, which are dominated by Proteobacteria and Acidobacteria, with Actinobacteria being the third most abundant phylum (Janssen, 2006). The Nullarbor soils are instead dominated by Actinobacteria, which is consistent with previous observations from Australian arid deserts (Holmes et al., 2000), the arid Tataouine Desert (Tunisia) (Chanal et al., 2006), and the hyper-arid Atacama Desert (Chile) (Neilson et al., 2012). The dominant OTU and key indicator species in all four meteorites demonstrated 98% 16S rRNA gene identity with *R. radiotolerans*. The parent genus, *Rubrobacter*, has previously been reported to comprise 2.6–10.2% of the OTU abundance in soil samples from within the arid desert of the Sturt National Park, New South Wales (Holmes et al., 2000). The two major OTUs in the meteorites, OTU1 and OTU2, were most similar to *R. radiotolerans* indicating species level diversity within the *Rubrobacter* exists in the Nullarbor soil samples, as has been previously identified in Sturt National Park (Holmes et al., 2000). *R. radiotolerans* is a “multi-extremophile,” which can survive at high temperature and high UV, and endure radiation damage (Egas et al., 2014). Ordinary chondritic meteorites are significantly more depleted in radioactive elements, such as Th and U, than most crustal rocks on Earth (Shinotsuka and Ebihara, 1997). It is unlikely that *R. radiotolerans* is present in Nullarbor soils and meteorites because there is a source of ionizing radiation in the meteorites. A more likely explanation is that this radiation resistance is a fortunate side effect of DNA repair strategies associated with adaptation to desiccation and/or oxidative stress (Egas et al., 2014) in arid environments like the Nullarbor Plain.

Without functional gene analysis, we cannot tell with certainty whether OTU1 and OTU2 (which share 98% and 96% rRNA 16S gene identity, respectively, to *R. radiotolerans*) indeed have these traits. Such adaptations would be beneficial in the semi-arid and desiccating conditions of the Nullarbor Plain and the arid interior of Australia. The similarity between the communities from Sturt National Park and the Nullarbor Plain may be due to aeolian processes (i.e., dust storms) that are known to transport microbial flora across Australia (De Deckker et al., 2014).

A key identifier species (i.e., OTU8 and OTU24) in the soil samples were most closely related to a known AOA, *Nitrosocosmicus franklandus*, and it is possible that these and other Thaumarchaeota identified in the Nullarbor soil samples are ammonia oxidizers, given that AOAs are common in that phylum (Pester et al., 2011). Thus, they may play an important role in nitrogen cycling in the Nullarbor soils. Few OTUs closely related to known Ammonia Oxidising Bacteria (AOB) (e.g., Beta- and Gammaproteobacteria) (Purkhold et al., 2000; Pester et al., 2011) were identified in this study. Also, a large proportion of the sequences from the soil samples were not closely related to any currently cultured species (e.g., the top 5 OTUs in soil sample SB were <90% similar to any cultured species); thus, there remains much to be uncovered about Nullarbor soil microbial communities in the future. A note worth considering is that the soils sampled were directly beneath the meteorites and may not be representative of Nullarbor soil in the open. The meteorites shield the underlying soil from sunlight and serve as moisture traps; this may have led to a bias toward hypolithic organisms resulting in fewer sampled phototrophs or xerophilic organisms due to this microenvironmental difference. The meteorites we sampled were also small, and of similar size to cobbles found on the deflationary gibber surface of the Nullarbor Plain, which suggests that hypolithic organisms should already be common in these soil samples. However, the deeper nature of the cored soil profile (∼2 cm) should mask any variation in microhabitat and reduce bias toward sampling hypolithic organisms.

### Comparison to Volcanic Rocks

We found similar abundances of Actinobacteria in meteorites collected from the Nullarbor Plain to those previously reported by other workers during studies of volcanic (basaltic) glasses (Kelly et al., 2010). Kelly et al. (2010) found that 43% of the sequences in basaltic glasses from Iceland were Actinobacteria followed by lesser abundances of Proteobacteria, Acidobacteria, and Cyanobacteria. They attributed colonization of basaltic glass to the liberation of bioessential elements (e.g., Fe, Mg, Ca) during weathering, which is rapid for basaltic glasses. This could create a nutritional advantage for certain organisms able to capitalize on the liberation of these cations. Meteorites commonly have a similar glassy coating called a ‘fusion crust.’ This crust is formed during ablative melting of the major silicate, sulfide and metal alloy minerals in meteors as the enter Earth’s atmosphere. The melt quenches to form a silicate glass that is rich in Mn, K, Na, and Al, with some Fe and Cr (Genge and Grady, 1999). Fusion crusts are weathered quickly due to their high reactivity with the atmosphere, surface and meteoric waters. As with basaltic glasses, oxidative weathering and reaction with carbonic acid in rain water combine to mobilize the elements needed to produce hygroscopic alteration minerals [e.g., carbonates, sulfates, Fe-(oxy)hydroxides and smectites] (Tait et al., 2017). Microbes that inhabit meteorites have been found just millimeters underneath fusion crusts in association with these alteration minerals (Tait et al., 2017) indicating that a similar process may be occurring in meteorites as has been observed in volcanic rocks in Iceland.

We did not find any significant trends between microbial ecology and the physical characteristics of meteorites (e.g., shock, weathering grade). Indeed all meteorites in this study were of the same chemical class of meteorite (i.e., they are L type ordinary chondrites) and come from the same parent body. It is not possible to distinguish between classes of ordinary chondrite (e.g., H, L, LL) by visual inspection in the field; thus, a higher number of samples may be needed to elucidate whether different microbial communities might colonize H or LL ordinary chondrites.

Understanding the chemical and physical properties of meteorites is important as they control the amount of porosity in the meteorite at any one time; porosity provides an upper limit for the amount of biomass in an endolithic community (Pontefract et al., 2016). Meteorites have variable amounts of porosity, which is retained from primary accretion of the parent body and modified by later impacts between asteroids (Wilkison et al., 2003). Although the amount of porosity in ordinary chondrites has no observed correlation with shock, there is large scatter in the data that may hide such a trend (Wilkison et al., 2003). In carbonaceous meteorites, the availability of primary porosity is inversely related to the amount of shock a meteorite has experienced (Tait et al., 2016). The opposite is true of impact basins on Earth, where crack networks from impacts are known to promote microbial diversity (Pontefract et al., 2016). Impacts do not adversely affect the bioavailability of elemental nutrients to microbial communities in shocked rocks on Earth (Pontefract et al., 2012). Given that similar impact process operated on meteorite parent bodies, it is likely that bioavailability of elemental nutrients will be similarly unaffected in meteorites. Mineral weathering is another important factor that will control microbial colonization of meteorites. For instance, reaction-driven cracking during precipitation of secondary minerals can increase porosity and permeability within meteorites. Contrastingly, internal porosity can also be filled by the oxidative weathering products of troilite and FeNi alloys (Bland et al., 2006), which may limit colonization of pore spaces (Tait et al., 2017). It is worth noting that Actinobacteria was also the dominant microbial phylum in a study of shocked terrestrial rock found in impact basins on Earth (Pontefract et al., 2016), implying this phylum may have members that excel in endolithic microenvironments.

## Conclusion

This study has found that microbial communities in samples of Nullarbor Plain soil are more diverse than those in meteorites, which are highly uniform in their species evenness and species richness despite having been found several kilometers apart and on soils with different microbial communities. Moreover, all meteorites were dominated by a single OTU classified as a member of the Actinobacteria, affiliating with *Rubrobacter radiotolerans*. The meteorites in this study are chemically similar L chondrites, but they have varied shock, weathering and thermal metamorphic histories. We could not discern, based on the small number of samples studied, whether these factors might contribute to development of different microbial populations. We did find OTUs affiliating with known iron and sulfur reducing genera, *Geobacter* and *Desulfovibrio*, but these were present in low abundance and did not contribute significantly to differences in community structure between meteorites and soils. Nonetheless, these organisms could potentially play a role in meteorite weathering. The chemical composition, and possibly the albedo of the meteorites, seems more likely to control community structure than the subtleties of their histories. This study has shown that microbes can exploit rock types and environments that are different from the soils that host parent communities. Specifically, once a community has taken hold in the new substrate, its structure will tend to reflect the environmental and chemical forcing of that habitat, rather than retain the structure of the parent soil community. This has consequences for any future sample return of exogenic meteorites from Mars as proposed by Tait et al. (2017). If meteorites returned from Mars were to contain evidence of a past putative biosphere, it could be that putative microorganisms in meteorites might not be indicative of the true complexity of the immediate environment.

## Author Contributions

This project was conceived of and executed by AWT. Design of methodology and significant conceptualisation was conducted by EG and AWT. AWT led the writing of the paper, with significant contributions by EG. AWT constructed figures and tables. DNA extraction was conducted by EG. Feedback with regards to methodology, techniques and interpretation was given by SW, AGT, and GS. All authors contributed to the discussion, interpretation and writing.

## Conflict of Interest Statement

The authors declare that the research was conducted in the absence of any commercial or financial relationships that could be construed as a potential conflict of interest.

## Footnotes

1 http://www.bom.gov.au/jsp/ncc/climate_averages/uv-index/index.jsp, accessed online May 2017.

2 http://www.bom.gov.au/climate/averages/tables/cw_018110.shtml, accessed online May 2017.

3 https://www.mothur.org/wiki/MiSeq_SOP, accessed online November 2016.
