# Supplementary material for: Microbial Populations of Stony Meteorites: Substrate Controls on First Colonizers
Source: Front Microbiol. 2017 Jun 30;8:1227. doi: 10.3389/fmicb.2017.01227 (PMC5492697; doi:10.3389/fmicb.2017.01227)
Supplement: Supplementary file 1 [file Table_1.DOCX]

**Supplementary Table 1| Linear Discriminant Analysis Effect Size (Lefse) Results.**

| ***Top 25 OTUs*** | |  |  |  |  |  |  |
| --- | --- | --- | --- | --- | --- | --- | --- |
| *OTU* | *%ID* | *#Accession* | *BLAST Identity* | *LogMaxMean^§^* | *Class^★^* | *LDA** | *p Value* |
| OTU1 | 98% | CP007514.1 | *Rubrobacter radiotolerans* | 5.174 | Meteorite | 4.818 | 0.034 |
| OTU2 | 96% | CP007514.1 | *Rubrobacter radiotolerans* | 4.693 | Meteorite | 4.370 | 0.034 |
| OTU3 | 98% | CP002408.1 | *Nitrososphaera gargensis* | 4.576 | - | - | - |
| OTU4 | 86% | DQ812549.1 | Chloroflexi Bacterium Ver9Iso1 | 4.700 | Soil | 4.427 | 0.034 |
| OTU5 | 99% | LN626276.1 | *Blastococcus sp.* | 4.425 | Meteorite | 3.926 | 0.034 |
| OTU6 | 100% | LN626270.1 | *Geodermatophilus pulveris* | 4.388 | Meteorite | 3.969 | 0.034 |
| OTU7 | 94% | AY234727.1 | *Blastococcus sp.* | 4.301 | - | - | - |
| OTU8 | 98% | KU290365.1 | *Nitrosocosmicus franklandus* | 4.056 | - | - | - |
| OTU9 | 90% | NR_125642.1 | *Kallotenue papyrolyticum* | 4.497 | Soil | 4.236 | 0.019 |
| OTU10 | 89% | NR_117433.1 | *Rubellimicrobium sp.* | 4.395 | - | - |  |
| OTU11 | 97% | KT581436.1 | *Patulibacter brassicae* | 3.844 | - | - |  |
| OTU12 | 91% | DQ812549.1 | Chloroflexi #Ver9Iso1 | 3.997 | Meteorite | 3.571 | 0.028 |
| OTU13 | 98% | NR_133858.1 | *Solirubrobacter phytolaccae* | 3.878 | - |  | - |
| OTU14 | 90% | NR_074501.1 | *Acidothermus cellulolyticus* | 4.053 | Soil | 3.789 | 0.019 |
| OTU15 | 99% | KU258211.1 | *Blastococcus jejuensis* | 4.000 | Meteorite | 3.553 | 0.034 |
| OTU16 | 98% | AB461094.1 | Nocardioidaceae #IK2_56P | 3.884 | - | - | - |
| OTU17 | 95% | KP326333.1 | *Sphingosinicella sp.* | 3.937 | - | - | - |
| OTU18 | 94% | NR_042722.1 | *Solirubrobacter pauli* | 3.980 | - | - | - |
| OTU19 | 99% | KX990261.1 | Frankiales (Unclassified) | 3.933 | Meteorite | 3.650 | 0.034 |
| OTU20 | 88% | NR_074501.1 | *Acidothermus cellulolyticus* | 3.758 | - | - | - |
| OTU21 | 94% | NR_043156.1 | *Deinococcus navajonensis* | 3.929 | Meteorite | 3.550 | 0.034 |
| OTU22 | 89% | KJ535408.1 | Candidatus Hydrogenedentes | 4.176 | - | - | - |
| OTU23 | 99% | NR_117640.1 | *Rubrobacter sp.* | 3.917 | Meteorite | 3.706 | 0.032 |
| OTU24 | 98% | KU290365.1 | *Nitrosocosmicus franklandus* | 3.964 | - | - | - |
| OTU25 | 91% | NR_118138.1 | *Gaiellaceae gaiella* | 4.147 | Soil | 3.887 | 0.028 |
|  |  |  |  |  |  |  |  |
| ***Possible Metal/Sulfur Cycling OTUs*** | | |  |  |  |  |  |
| *OTU* | *%ID* | *#Accession* | *BLAST Identity* | *LogMaxMean^§^* | *Class^★^* | *LDA** | *p Value* |
| OTU71 | 100% | CP014963.1 | *Geobacter anodireducens* | 3.421 | - | - | - |
| OTU264 | 100% | NR_074979.1 | *Geobacter lovleyi* | 2.864 | - | - | - |
| OTU501 | 100% | KU921226.1 | *Desulfovibrio desulfuricans* | 2.489 | - | - | - |
| OTU712 | 100% | KU201952.1 | *Desulfovibrio sp.* | 2.196 | - | - | - |
| OTU730 | 100% | EU190359.2 | *Desulfovibrio intestinalis* | 2.237 | - | - | - |
| OTU884 | 99% | NR_075011.1 | *Geobacter metallireducens* | 2.116 | - | - | - |
| OTU1164 | 99% | HQ395074.1 | *Desulfovibrio vulgaris* | 2.050 | - | - | - |
| OTU1518 | 100% | NR_028775.1 | *Geobacter thiogenes* | 1.860 | - | - | - |
| OTU1812 | 98% | CP002297.1 | *Desulfovibrio vulgaris* | 1.766 | - | - | - |
| OTU2077 | 99% | AF443593.1 | *Desulfomicrobium sp* | 1.503 | - | - | - |
| OTU2350 | 98% | LC008330.1 | *Geobacter sp.* | 1.591 | - | - | - |
| OTU2784 | 97% | GU176294.1 | *Desulfovibrio sp* | 1.725 | - | - | - |
| OTU2886 | 96% | LC186051.1 | *Desulfovibrio sp* | 1.445 | - | - | - |
| OTU4867 | 97% | LC008330.1 | *Geobacter sp.* | 1.202 | - | - | - |
| OTU4868 | 96% | LC186051.1 | *Desulfovibrio sp* | 1.230 | - | - | - |
| OTU7045 | 99% | EU190359.2 | *Desulfovibrio sp* | 1.178 | - | - | - |
| OTU7098 | 95% | KJ459867.1 | *Desulfovibrio desulfuricans* | 1.418 | - | - | - |
| OTU10078 | 97% | LC008330. | *Geobacter sp.* | 0.761 | - | - | - |
| OTU10079 | 96% | NR_075011.1 | *Geobacter metallireducens* | 0.743 | - | - | - |
| OTU12162 | 96% | KU201952.1 | *Desulfovibrio sp.* | 1.157 | - | - | - |
| OTU12175 | 98% | EF055877.1 | *Desulfovibrio sp.* | 1.157 | - | - | - |
| OTU12259 | 98% | NR_029364.2 | *Desulfovibrio longreachensis* | 1.002 | - | - | - |
|  |  |  |  |  |  |  |  |
| * LDA = Linear Discriminant Analysis, is the effect size. | | | |  |  |  |  |
| § LogMaxMean = Is the log of the greatest class mean. | | | |  |  |  |  |
| ★ Class = Refers to either the meteorite or soil sample type being examined by Lefse. | | | |  |  |  |  |
